# Long-standing necrobiotic xanthogranuloma limited to the skin: A case report

**DOI:** 10.1177/2050313X211057929

**Published:** 2021-11-09

**Authors:** Anne-Sophie Smilga, Mona Lavoie, Sungmi Jung, Ariane Schreiber

**Affiliations:** 1 Dermatology, Université Laval, Québec, QC, Canada; 2 Pathology, Hôtel-Dieu de Lévis, Lévis, QC, Canada; 3 Pathology, McGill University, Montréal, QC, Canada; 4 Dermatology, Hôtel-Dieu de Lévis, Lévis, QC, Canada

**Keywords:** Dermatology, inflammatory dermatoses, pathology

## Abstract

Necrobiotic xanthogranuloma is a rare non-Langerhans cell histiocytosis with a known association with monoclonal gammopathies and malignant conditions. There is a lack of consensus regarding the management of necrobiotic xanthogranuloma. In this case report, the patient is diagnosed with a long-standing necrobiotic xanthogranuloma limited to the skin. Although all initial investigations were reassuring, the patient remains at a higher risk of developing a malignant condition. The goal of the authors is to highlight the need for clearer investigation and follow-up guidelines.

## Introduction

Necrobiotic xanthogranuloma (NXG) is a rare non-Langerhans cell histiocytosis. It is a progressive multisystem histiocytic disease that can lead to organ dysfunction and death. The average age of onset is in the sixth decade.^1,2^ Extensive investigations are considered needed at the time of diagnosis because of the well-described association with monoclonal gammopathies and malignant conditions. There is, however, a lack of official consensus regarding the management of NXG.

We report a case of long-standing NXG limited to the skin. Our aim is to highlight the need for clearer investigation and follow-up guidelines.

## Case report

The patient is a 56-year-old Caucasian man with no pertinent past medical history. The reason for consultation was “recurrent eyelid dermatitis.” Upon questioning, the patient reports a 12-year history of oedematous eyelid lesions, describing occasional flares with pruritus. The patient noticed a slight amelioration in the past 4 months with the use of hydrocortisone valerate 0.2% cream twice daily. No other treatments were attempted. The patient additionally reports an occasional burning sensation in the eyes but denies any other ocular symptoms. He also denies any other skin and/or mucous membrane lesions. The review of systems is negative.

**Figure 1.**
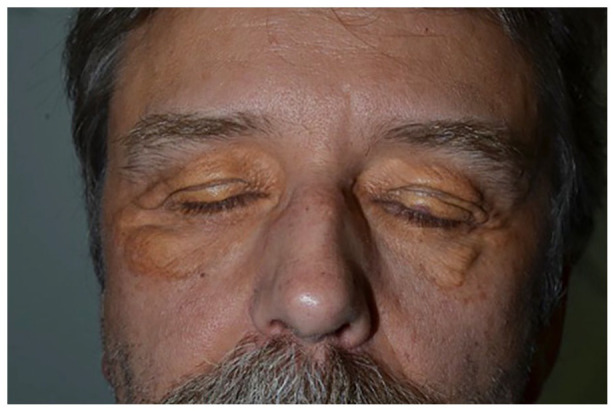
Confluent, yellow-brown plaques on the upper and lower eyelids, bilaterally. Non-palpable purpura also noted on the left upper eyelid.

**Figure 2.**
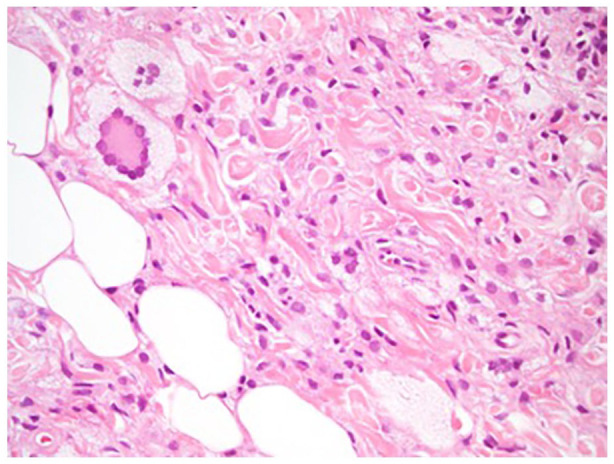
Touton type giant cells. Hematoxylin and eosin stain. 40× magnification.

On physical examination, there are confluent, yellow-brown plaques on the upper and lower eyelids, bilaterally. Non-palpable purpura is also noted on the left upper eyelid (Figure 1). There is no macroglossia. The ophthalmic examination is normal. An incisional punch biopsy (right lower eyelid) confirmed the diagnosis of NXG. Histopathology indeed shows a diffuse xanthomatized histiocytic infiltrate focally admixed with lymphocytes throughout the dermis into the subcutis. There are frequent Touton type giant cells noted (Figure 2). Laboratory investigations were all normal (complete blood count, renal panel, liver panel, lipid panel, thyroid-stimulating hormone, erythrocyte sedimentation rate, C-reactive protein, antinuclear antibody, rheumatoid factor, immunoglobulins, lactate dehydrogenase, serum protein electrophoresis and immunofixation, and urine protein electrophoresis). Also normal were the chest X-ray, chest–abdomen–pelvis computed tomography, and bone scan. A bone marrow biopsy was not indicated as per the hematology–oncology consultation.

The patient decided to only continue with his hydrocortisone valerate 0.2% cream twice daily. Other considered treatments were stronger topical corticosteroids, systemic corticosteroids, intravenous immunoglobulin, and thalidomide. Two years later, the patient’s condition is stable with no additional findings. There is no evidence of paraproteinemia.

## Discussion

Cutaneous lesions are present in virtually all NXG cases. Classically, multiple, asymptomatic, indurated, yellow-to-orange papules, and plaques and/or nodules are found in a periorbital distribution. Other reported features include ulceration, telangiectasias, atrophy, and induration. The remainder of the face, trunk, and proximal extremities can be involved, although less frequently. New skin lesions can also develop within scars. The eyes are the leading site of extracutaneous involvement, with up to half of the patients having ophthalmic manifestations. Other sites are the gastrointestinal tract, liver, heart, lungs, lymphoreticular system, parotid glands, brain, and muscles.^1,2^

There is a well-described association between NXG and monoclonal gammopathies, especially of the immunoglobulin G (IgG)-κ type. In a recent multicenter cross-sectional study and systematic review of NXG, paraproteinemia was detected in 82.1% of the patients. A malignant condition was also identified in 25.1% of the patients, with multiple myeloma, lymphoma, and leukemia being the most common ones.^2–4^ This underlies the need for clear investigation guidelines at the time of diagnosis as well as on follow-up care. In the case of our patient, there is an uncertainty regarding which investigations to repeat and when. Indeed, although all initial investigations were reassuring, the patient remains at a higher risk of developing a malignant condition.

There is also a lack of consensus regarding the optimal treatment approach and the urgency of implementing it. No controlled clinical studies are available regarding the treatment of NXG. Treatment options include topical and systemic corticosteroids, thalidomide, alkylating agents (chlorambucil, melphalan, and cyclophosphamide), antimalarials, high-dose intravenous immunoglobulin, rituximab, surgery, radiation therapy, psoralen and ultraviolet A radiation (PUVA) therapy, laser therapy, and plasmapheresis.^2,5–7^ Intravenous immunoglobulin appears particularly effective.^2,7^ Newer therapies await future studies.^2^

## References

[1] Bolognia JL Schaffer JV Cerroni L , et al. Non-Langerhans cell histiocytoses. In: Bolognia JL Schaffer JV Cerroni L , et al. (eds) Dermatology. Edinburgh: Elsevier, 2018, pp. 1614–1633.

[2] Nelson CA Zhong CS Hashemi DA , et al. A multicenter cross-sectional study and systematic review of necrobiotic xanthogranuloma with proposed diagnostic criteria. JAMA Dermatol 2020; 156(3): 270. 31940000 10.1001/jamadermatol.2019.4221 PMC6990734

[3] Omarjee L Janin A Etienne G , et al. Necrobiotic xanthogranuloma: a paraneoplastic skin lesion of haematological malignancies? Eur J Dermatol 2018; 28(3): 384–386. 29619997 10.1684/ejd.2018.3256

[4] Szalat R Pirault J Fermand JP , et al. Physiopathology of necrobiotic xanthogranuloma with monoclonal gammopathy. J Intern Med 2014; 276(3): 269–284. 24428816 10.1111/joim.12195 PMC4279948

[5] Al-Niaimi FA Dawn G Cox NH . Necrobiotic xanthogranuloma without paraproteinaemia: marked improvement with psoralen ultraviolet A treatment. Clin Exp Dermatol 2010; 35(3): 275–277. 19663852 10.1111/j.1365-2230.2009.03447.x

[6] Miguel D Lukacs J Illing T , et al. Treatment of necrobiotic xanthogranuloma—a systematic review. J Eur Acad Dermatol Venereol 2016; 31(2): 221–235. 27436448 10.1111/jdv.13786

[7] Olson RM Harrison AR Maltry A , et al. Periorbital necrobiotic xanthogranuloma successfully treated with intravenous immunoglobulin. Case Rep Ophthalmol 2018; 9(1): 70–75. 29643785 10.1159/000485913 PMC5892313

